# Retraction Note: LncRNA AK023391 promotes tumorigenesis and invasion of gastric cancer through activation of the PI3K/Akt signaling pathway

**DOI:** 10.1186/s13046-026-03804-5

**Published:** 2026-08-08

**Authors:** Yanxia Huang, Jing Zhang, Lidan Hou, Ge Wang, Hui Liu, Rui Zhang, Xiaoyu Chen, Jinshui Zhu

**Affiliations:** 1 Department of Gastroenterology, Shanghai Jiao Tong University Affiliated Sixth People’s Hospital, No. 600 Yishan Road, Shanghai, 200233 China; 2 Department of Gastroenterology, Shanghai Ninth People’s Hospital, Shanghai Jiao Tong University School of Medicine, Shanghai, China

**Retraction Note: J Exp Clin Cancer Res 36, 194 (2017)**

**https://doi.org/10.1186/s13046-017-0666-2**

The Editor-in-Chief has retracted this article. Concerns were raised regarding a number of figures, specifically;

- Figure 4: portions of the Merge panels in the HGC-27 row appear to overlap;
- Figure 5b: portions of the HGC-27 panels appear to overlap;
- Figures 5b and 5c: a portion of the SGC-7901/si-AK023391 panel in Fig. 5b and a portion of the SGC-7901/Vector panel in Fig. 5c appear to overlap after a rotation;
- Figure 8e: the AKT/HGC-27 and AKT/AGS bands appear to overlap;
- Figures 8e and 8f: the p-FOXO3a/SGC-7901 band in Fig. 8e and c-myb/SGC-7901 band in Fig. 8f appear to overlap after a rotation.

The authors provided some source data on request from the Publisher, but this was insufficient to address the concerns raised. The Editor-in-Chief therefore no longer has confidence in the reliability of the data reported in the article.

None of the authors responded to any correspondence regarding this retraction.

